# Electrochemistry of Tetrathiafulvalene Ligands Assembled on the Surface of Gold Nanoparticles

**DOI:** 10.3390/molecules27217639

**Published:** 2022-11-07

**Authors:** Jiří Janoušek, Jiří Rybáček, Miloš Buděšínský, Lubomír Pospíšil, Irena G. Stará, Ivo Starý

**Affiliations:** 1 Institute of Organic Chemistry and Biochemistry of the Czech Academy of Sciences, Flemingovo nám. 542/2, 160 00 Prague, Czech Republic; 2 J. Heyrovský Institute of Physical Chemistry of the Czech Academy of Sciences, Dolejškova 2155/3, 182 23 Prague, Czech Republic

**Keywords:** gold nanoparticles, tetrathiafulvalene, synthesis, TEM, spectroscopy, AC voltammetry

## Abstract

The synthesis of a tetrathiafulvalene (TTF) derivative, *S*-[4-({4-[(2,2′-bi-1,3-dithiol-4-ylmethoxy)methyl] phenyl}ethynyl)phenyl] ethanethioate, suitable for the modification of gold nanoparticles (AuNPs), is described in this article. The TTF ligand was self-assembled on the AuNP surface through ligand exchange, starting from dodecanethiol-stabilized AuNPs. The resulting modified AuNPs were characterized by TEM, UV-Vis spectroscopy, and electrochemistry. The most suitable electrochemical method was the phase-sensitive AC voltammetry at very low frequencies of the sine-wave perturbation. The results indicate a diminishing electronic communication between the two equivalent redox centers of TTF and also intermolecular donor–acceptor interactions manifested by an additional oxidation wave upon attachment of the ligand to AuNPs.

## 1. Introduction

Tetrathiafulvalene (TTF) derivatives have become well-known for their characteristic properties, namely their strong electron-donating character, geometrical flexibility, and well-defined reversible electrochemical oxidation process comprising two one-electron stages, TTF^●+^ and TTF^2+^. These unique features led to successful applications in various fields of science, such as supramolecular chemistry, organic electronics, and electrochemistry [1,2].

Gold nanoparticles (AuNPs) represent a well-established type of nanomaterial, which is easily accessible in different shapes and sizes and can be tailored to a wide range of properties through suitable surface modifications [3]. Small metallic particles must be stabilized either sterically (by neutral ligands) or electrostatically (e.g., by negatively charged citrate anions) to avoid aggregation [4]. Modern procedures for the preparation of AuNPs rely on the method developed by Turkevich in 1951 [5], often employing citrate as the reducing agent [6]. Here, the size of the resulting AuNPs depends on the ratio of citrate to the gold precursor.

In metallic nanoparticles, collective modes of motion of the electron gas can be excited upon interaction with incident light [7], giving rise to a “surface plasmon band” (SPB) in the visible region of the absorption spectrum (around 530 nm) for AuNPs larger than 2 nm [8]. The position and shape of the SPB strongly depends on nanoparticle shape, size, ligand, environment, and polydispersity and thus serves as an efficient characterization tool in addition to direct visualization by TEM.

Gold nanoparticles yield exceptional electron transfers (ET), which are controlled by quantum effects [9]. As an example, AuNP nanocatalysts can change the number of electrons involved in the oxidation−reduction reaction of eosin. Reduction by sodium borohydride involves a two-electron transfer, whereas reduction by AuNPs is a multi-step single-electronic reaction. The kinetics was monitored by the plasmonic band decay [10] and showed distinct differences compared to the ETs observed on macroscopic surfaces of electrodes. The modification of AuNPs by electron-donating or electron-accepting ligands can change their self-organization. Surface-bound ligands may also affect the respective ET processes. Thus, ligand molecules containing a π-electron system can show a donor–acceptor interaction with the gold surface. Applications include catalysis, materials science, and biology. For example, a thin film of conjugated polymer with embedded AuNPs exhibited negative differential resistance and memory behavior with a high ON-OFF current ratio [11]. Embedding AuNPs in lipid monolayers was explored for biological applications [12]. Using AuNPs in flexible poly(dimethylsiloxane) cross-linked by metal-ligand coordination systems influenced the conductivity of the resulting material [13]. Numerous applications of AuNPs have been reviewed [14]. Experimental attempts also include research on single AuNPs [15,16].

To date, only a few examples of electrochemical studies of AuNPs decorated with TTF units have been described in the literature. However, AC voltammetry has not been employed so far. Fujihara and colleagues created a stable gold electrode modified with AuNPs covered or interlinked by TTF thiols or tetrathiols [17]. Guo et al. studied using cyclic voltammetry AuNP systems with the TTF derivatives being directly linked to the gold surface through the sulfide bond, omitting any spacers [18,19]. The electrochemical behavior of TTF on other NP materials, such as PdNP [20] or PbS NP [21], was also studied.

**Figure 1:**
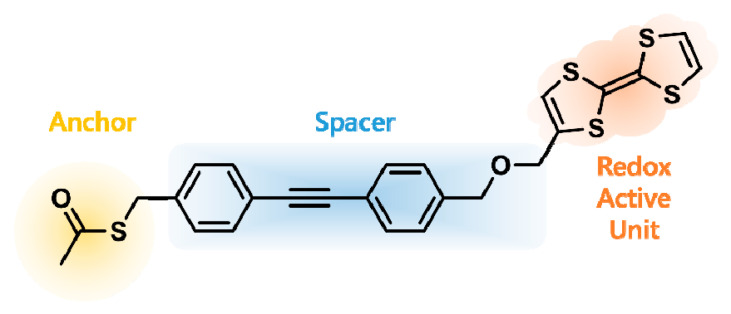
Structure of the ligand **1** used for AuNP modification.

In this communication, we describe the use of 10 nm AuNPs functionalized with the rod-shaped TTF derivative **1** (Figure 1) as a model to study the electrochemical properties of redox-active self-assembled ligands on a curved nanoparticle surface. Based on this design, the strong but mobile binding mode between the ligand and the AuNPs through the S–Au bond and the semi-rigid spacer should ensure optimum compact packing in thermodynamic equilibrium. We looked at the effect of such a spatial arrangement of the TTF units on their redox properties. Our approach uses a non-metallic glassy carbon electrode instead of a working electrode made of Pt or Au. Unlike other authors, we did not apply long-dip deposition.

## 2. Results

### 2.1. Synthesis of TTF Ligand ***1***

**Scheme 1:**
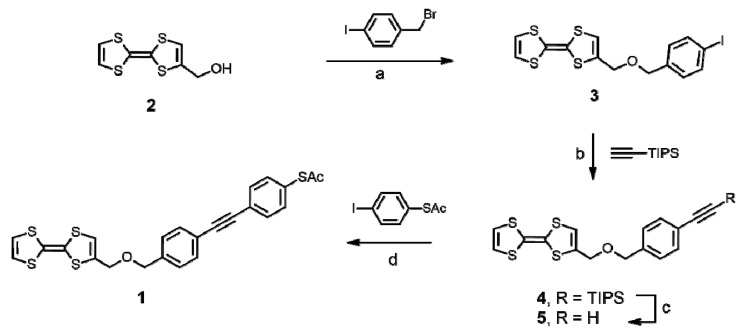
Synthesis of ligand **1**. Conditions: (a) NaH (3.0 equiv.), THF, rt, 15 min and then 4-iodobenzyl bromide (1.1 equiv.), THF, rt, 3 h, in the dark, 77%; (b) ethynyltriisopropylsilane (2.0 equiv.), Pd(PPh_3_)_4_ (2.3 mol%), CuI (14 mol%), ^i^Pr_2_NH, rt, 12 h, 82%; (c) Bu_4_NF (1.5 equiv.), THF, rt, 20 min, used without purification in the next step; (d) 4-iodophenyl thioacetate (2.2 equiv.), Pd(PPh_3_)_4_ (4 mol%), CuI (20 mol%), Et_3_N, THF, 35 °C, 1 h, ultrasonic bath, 81%.

Scheme 1 shows the straightforward synthetic route employed in the synthesis of ligand **1,** starting from the known hydroxymethyl TTF derivative **2 [22]**. A nucleophilic substitution reaction with commercially available 4-iodobenzyl bromide produced TTF iodide **3**, which, upon Sonogashira coupling with trimethylsilyl acetylene, afforded aromatic alkyne **4**. After desilylation by tetrabutylammonium fluoride, the terminal triple bond was coupled to *S*-(4-iodophenyl) thioacetate to obtain the target TTF ligand **1**. A detailed structure analysis of **1** and its precursors is provided in experimental section and supplementary material (Figure S1).

### 2.2. Surface Modification of AuNPs

First, dodecanethiol-stabilized AuNPs (C_12_H_23_S@AuNPs) with a ~10 nm diameter were prepared following a literature procedure [23]. The TEM image of the C_12_H_23_S@AuNP monolayer and the corresponding size-distribution histogram can be found in the Supplementary Material (Figure S2). Then, the acetyl group was cleaved from ligand **1** by the action of methanolic tetrabutylammonium hydroxide and the resulting solution was mixed with ethanolic solution of C_12_H_23_S@AuNPs. The ligand exchange was completed within 2 days at room temperature in the dark. Solubilization of the new **1**@AuNPs could be achieved by sonication in dichloromethane, chloroform, or 1, 2-dichloroethane (DCE).

**Figure 2:**
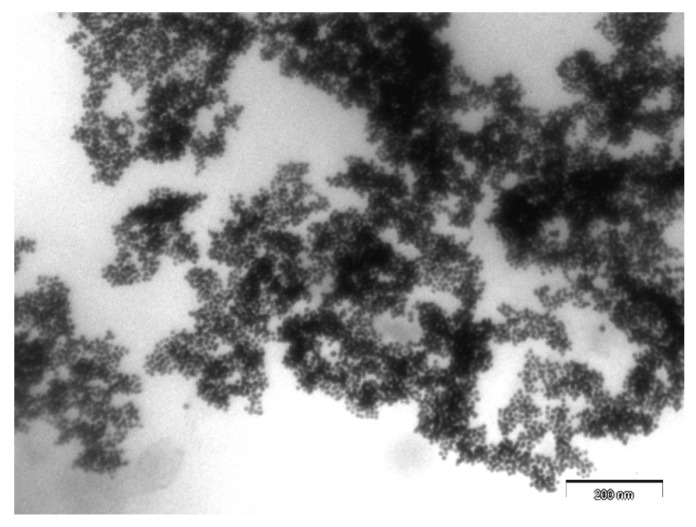
TEM image (100,000 × magnification) of aggregates of **1**@AuNPs.

Compared to the uniform monolayers formed at the water–air interface by C_12_H_23_S@AuNPs, the **1**@AuNP nanomaterial was much more prone to aggregation, as illustrated by the TEM image (Figure 2).

### 2.3. UV-Vis Spectroscopy

**Figure 3:**
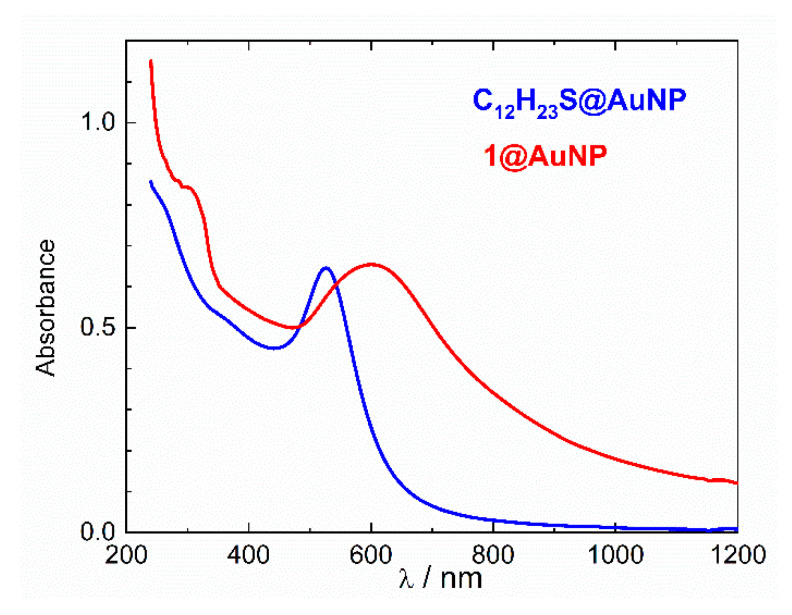
UV-Vis spectra of C_12_H_23_S@AuNPs and **1**@AuNPs in chloroform.

The position of the characteristic SPB for AuNPs is very sensitive to the character of the ligand attached to its surface. Due to the donor–acceptor interaction between the π-aromatic system of the electron-donating ligand **1** and the surface gold atoms of the AuNPs, a bathochromic shift of the plasmonic band was induced in **1**@AuNPs compared to the C_12_H_23_S@AuNPs. Depending on the solvent, the difference was 73 to 103 nm. Figure 3 shows the comparison of the UV-Vis spectra of C_12_H_23_S@AuNPs (SPB at 527 nm) and **1**@AuNPs (SPB at 600 nm) in chloroform. A broader SPB together with higher absorption in the high-wavelength region (~800 nm) indicate increased light scattering caused by partial aggregation of the surface-modified AuNPs.

### 2.4. Electrochemistry

Due to insufficient sensitivity, attempts to detect the redox processes of **1**@AuNPs by cyclic voltammetry were not successful due to insufficient sensitivity. Large capacitive and very small faradaic currents could not be accurately resolved. The electric conductivity of samples is achieved by indifferent and redox-silent electrolytes. The electrode charge attracts ions of the electrolyte, thus forming a surface double layer as a capacity *C*. Charging of the double-layer capacity was a common problem for the analysis of traces of various compounds in the environment, medicine, etc. The solution came from a superposition of small amplitude rectangular pulses on the DC voltage scan. The modification of voltage-scanning methods (differential pulse polarography and square-wave voltammetry) was based on different current time decays of charging and faradaic currents. Any change in the DC potential leads to the current charging of *C* at the electrode surface. The process is fast and the charging current exponentially decays with time. Faradaic currents decay slowly with the square root of time. Pulse methods read the current at each pulse end, which is free of the charging contribution. This greatly improves the detection limits of trace analysis. Information on the changes in the double-layer *C* are lost. Research projects often need information on reactant or product adsorption, monolayer formation, the phase condensation of adsorbed films, and corrosion inhibitors, which are all sensitively reflected by *C* changes. An alternative is the application of a small sine-wave signal on the DC scanning voltage. Here, the time delay read-out of pulse methods is replaced by two read-outs at different phase shifts with respect to the applied sine-wave “perturbation”. The instrument uses two channel outputs, one tuned in-phase (0° phase shift) with respect to sine-wave input and the second one shifted 90° with respect to the superimposed AC signal. The instruments are called “locked-in” or “phase-sensitive” amplifiers. An antiquated name, not used anymore, was “vector polarography”. The measurements yield two curves from two output channels of the amplifier. The AC methods find important application in the research on the mechanism of the kinetics of electron transfer rates and coupled chemical and catalytic reactions. The great advantage is the availability of a large scale of time constants for the characterization of the various redox systems. The time constants of AC methods can cover five decades of sine frequencies from 1 Hz to 10 kHz.

The application of the phase-sensitive AC voltammetry operating at low frequencies in the range 1.6 Hz to 16 Hz turned out to be the method of choice for our system. The amplitude of the superimposed AC signal was 10 mV (p-p). Such low frequencies used here are rather exceptional for this method. They help to minimize the imaginary admittance component of the blank, which is proportional to the frequency. The real and imaginary faradaic components superimposed over the blank decrease with the square root of the frequency; hence, the low frequency was used. These phase properties of the electrode admittance make it possible to find an optimum for the faradaic and background separation. Here, we report faradaic components already separated from the charging vectors.

**Figure 4:**
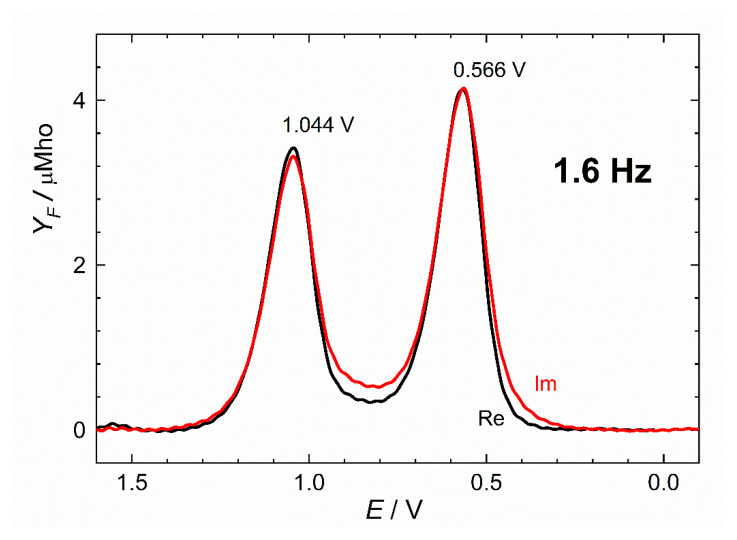
AC voltammogram at 1.6 Hz shows the faradaic part of the admittance vector of ligand **1** (DCE/Bu_4_NPF_6_). The real (Re) and imaginary (Im) components are black and red curves, respectively.

Figure 4 shows a typical AC voltammogram of ligand **1** in dichloroethane with an expected redox behavior of TTF. Two AC faradaic admittance maxima with real and imaginary components of equal height prove to be reversible electron transfers (ET). The separation of the two one-electron ETs confirms the electronic communication between the two structurally equal redox centers through electrostatic interaction [24,25,26,27]. Figure 4 confirms that the structure of **1**, used for AuNP modification, does not deteriorate the redox activity of the TTF substituent.

**Figure 5:**
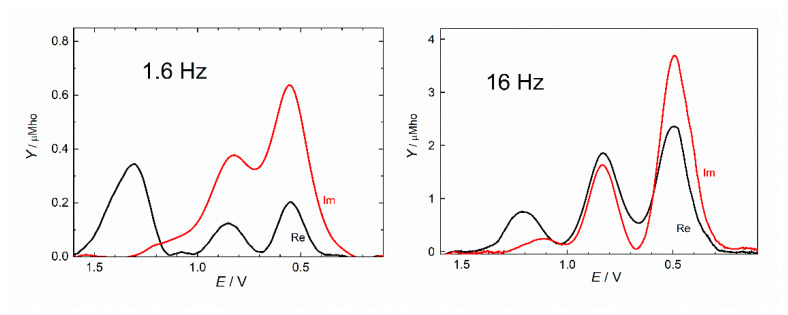
AC voltammogram at 1.6 Hz (**left**) and 16 Hz (**right**). The faradaic part of the admittance vector of **1**@AuNPs (DCE/Bu_4_NPF_6_). The real (Re) and imaginary (Im) components are black and red curves, respectively.

The values of Δ*E*^0′^ for various redox systems can be found in a wide range from 10 mV to 800 mV and also depend on the dielectric permittivity of the solvent and the type of counterions [28,29]. Ligand **1** in solution produces the value of Δ*E*^0′^ = 0.478 V. Surface-modified **1**@AuNPs yield Δ*E*^0′^ = 0.304 ± 5 (Figure 5). 

To exclude the interference of any redox processes originating from the thiol-coated AuNPs themselves, an analogous phase-sensitive AC voltammetry (1.6 Hz, DCE/Bu_4_NPF_6_) was performed on the C_12_H_23_S@AuNP material, which showed no faradaic processes (Figure S3, Supplementary Material).

The primary electron transfer (ET) is indeed from ligand **1**, which adsorbs on gold particles. This is evidenced by the ratio of the imaginary faradaic maximum Y^″^_F_ and the real faradaic maximum Y^′^_F_. An ordinary non-complicated ET is characterized by Y^″^_F_ ≤ Y^′^_F_. Equality holds for a reversible ET, whereas non-equality indicates a kinetically controlled ET. The rate of ET at a given DC potential is:

(1) $$k\left(\mathrm{ET}\right)=\;\frac{Y_{F}^{"}}{Y_{F}^{\prime }-Y_{F}^{\prime \prime }}\sqrt{2\omega D}$$

where *D* is the diffusion coefficient and ω is the angular frequency of the superimposed sine wave. An extensive mathematical background can be found in many text books [30].

**Figure 6:**
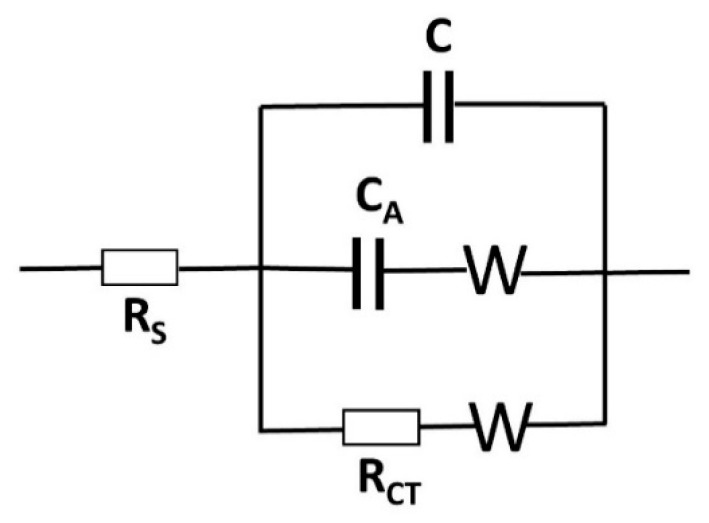
Equivalent electric circuit for the electrode impedance in case of adsorption of reactant. R_S_ is the solution resistance, *C* is the double-layer capacity, *C*_A_ is the adsorption “pseudocapacity”, and R_CT_ is the charge transfer related to Equation (1). Ws are Warburg impedances representing the influence of diffusion. The working electrode is on the right lead of the circuit and the counter electrode on the left side.

Here, one can observe a large reverse relation, Y^′^_F_ ≤ Y^″^_F_. The oxidation of **1**@AuNPs occurs at the positive electrode potential. After the first ET, the attached ligand becomes positively charged and electrostatic interaction between the electrode and **1**@AuNPs eliminates the adsorption effects for the second ET (Figure 5, right). Many adsorbing organic redox systems show the coupling of faradaic and double-layer charging. This coupling adds a contribution of an “adsorption pseudo-capacitance” *C*_A_ to the imaginary part Y^″^_F_ (Figure 6).

The a priori non-separability of these two processes was firstly reported by Delahay [31]. The mathematical solution of the problem shows that kinetic and double-layer parameters are always mutually multiplied, which results in their non-separability [32]. The effect is especially important for very fast ETs. Simulations found limits in the case of irreversible ETs [33]. This coupling effect is the reason why we observe, for the first ET, the relation Y^′^_F_ ≤ Y^″^_F_ as mentioned above for the first ET.

The origin of the third (broader) maximum of the faradaic real component Y^′^_F_ at the most positive potentials (Figure 5) is somewhat more difficult to explain. In principle, it could arise from the interaction of ligand **1** with the surface gold atoms of the AuNPs, for example, an oxidation accompanied by dissociation, but a similar process would have been observed for C_12_H_23_S@AuNPs as well, which was not the case, vide supra. Thus, we conclude that the more energetic process (~1.2 V) is most likely the result of intermolecular interactions taking place between two or more molecules of ligand **1** brought into close contact by the self-assembly on the curved AuNP surface. Further investigations are needed to elucidate a detailed mechanism.

## 3. Materials and Methods

^1^ H and ^13^ C NMR spectra were obtained on a Bruker Advance 600 (600.13 MHz for ^1^ H and 150.92 MHz for ^13^ C) spectrometer using acetone-*d_6_* as the solvent. The spectra are referenced to a residual solvent signal (2.05 ppm for ^1^ H and 29.84 ppm for ^13^ C) and the chemical shifts are reported in δ scale (ppm) and coupling constants *J* in Hz. The EI-MS spectra were acquired on ZAB–EQ (VG Analytical) and the m/z peaks are reported together with their relative intensities: the TOF-ESI MS spectra on LCQ Fleet (Thermo Fisher Scientific, Waltham, MA, USA) and the HR-ESI-MS spectra on LTQ Orbitrap XL (Thermo Fisher Scientific, Waltham, MA, USA) instruments. The the UV-Vis spectra were recorded with a Perkin Elmer Lambda 19 spectrophotometer using 1 cm cuvettes and spectroscopic-grade CHCl_3_. The IR spectra were measured in a KBr cell in CHCl_3_ solution (or in a KBr pellet as indicated) on a Nicolet 6700 FT-IR spectrometer (Thermo Fisher Scientific, Waltham, MA, USA) equipped with a standard mid-IR source, a KBr beam-splitter, a DTGS detector, and a cell compartment purged by dry nitrogen. Samples for the transmission electron microscopy (TEM) were prepared by transfer of monolayers from the water surface onto a carbon-coated copper grid (Custom Coated Grids 300 mesh regular, Pyser-SGI). Pictures were taken with a JEOL JEM 1200EX instrument working at 60 kV and were analyzed bythe program ImageJ [34], as described earlier [35]. Electrochemical measurements used a fast non-commercial potentiostat and a lock-in phase-sensitive amplifier (Stanford Research model SRS830, Stanford, CA, USA). The instruments were interfaced to a personal computer via an IEEE interface card (model PCL-848, PC-Lab, Advantech, Danvers, MA, USA) and a data acquisition card (PCL-818, Advantech, Danvers, MA, USA) using 12-bit precision for A/D and D/A conversion. A three-electrode electrochemical cell was used. The reference electrode, Ag|AgCl|1 M LiCl, was separated from the test solution by a salt bridge. The working electrode was a glassy carbon minidisc of 0.5 mm in diameter sealed in a glass capillary. The solvent was 1,2-dichloroethane. The auxiliary electrode was a platinum wire. Oxygen was removed from the solution by passing a stream of argon saturated with vapors of the solvent. All measurements were obtained at room temperature. Samples of functionalized AuNPs were sonicated in dichloroethane before electrochemical measurement. TLC was performed on Silica gel 60 F_254_-coated aluminum sheets (Merck) and spots were detected by the solution of Ce(SO_4_)_2_·4H_2_O (1%) and H_3_P(Mo_3_O_10_)_4_ (2%) in sulfuric acid (10%). Flash chromatography was performed on Silica gel 60 (0.040–0.063 mm, Merck) using glass columns. Diisopropylamine and triethylamine were distilled from calcium hydride under nitrogen and degassed by three freeze-pump-thaw cycles before use; tetrahydrofuran was freshly distilled from sodium/benzophenone under nitrogen. All commercially available solvents, catalysts, and reagent-grade materials were used as received. The starting material, 2,2′-bi-1,3-dithiol-4-ylmethanol (**2**), was synthesized according to the literature procedure [22].

Compound **3***:* A suspension of hydroxymethyl-TTF compound **2** (272 mg, 1.16 mmol) and sodium hydride (60% suspension in mineral oil, 140 mg, 3.50 mmol, 3.0 equiv.) in dry THF (10 mL) was stirred at rt for 15 min. Then, a solution of 4-iodobenzyl bromide (379 mg, 1.28 mmol, 1.1 equiv.) in THF was added and the reaction mixture was stirred at rt for 3 h in the dark. Upon addition of 2M HCl (20 mL) the mixture was extracted with CH_2_Cl_2_ (3 × 40 mL) and organic phase was dried over anhydrous MgSO_4_ and evaporated to dryness in vacuum. The crude product was purified by column chromatography on silica gel (hexane-acetone-ether 70:15:15) to obtain orange-yellow crystals of product **3** (402 mg, 77%). M.p. 95–96 °C (hexane). ^1^ H NMR (600 MHz, acetone-*d_6_*): 4.37 (d, *J* = 1.1, 2 H), 4.52 (d, *J* = 0.6, 2 H), 6.60 (t, *J* = 1.1, 1 H), 6.62 (d, *J* = 0.4, 2 H), 7.17–7.23 (m, 2H), 7.71–7.76 (m, 2 H). ^13^C NMR (150 MHz, acetone-*d*_6_): 67.83, 71.54, 93.38, 118.09, 120.34, 120.44, 130.70, 135.39, 138.29, 139.02, two quaternary carbon signals (TTF) could not be detected. HRMS (EI+): for C_14_H_11_OS_4_I calcd. 449.8738, found 449.8743. MS (EI+): 450 (M^+•^, 100), 324 (7), 234 (37), 232 (21), 231 (7), 218 (39), 216 (55), 202 (7), 173 (8), 167 (7), 116 (6), 107 (7), 90 (10), 89 (7), 79 (12), 78 (11), 77 (7), 76 (5). IR (KBr): 3433 w, 3063 w, 2973 w, 2940 w, 2889 w, 2856 w, 2843 w, 2788 w, 2763 w, 2739 w, 2687 w, 2647 w, 2593 w, 2537 w, 2449 w, 2403 w, 2361 w, 2343 w, 2188 w, 2095 w, 2072 w, 2046 w, 1974 w, 1911 w, 1892 w, 1846 w, 1792 w, 1635 w, 1623 w, 1589 w, 1578 w, 1551 w, 1540 w, 1517 w, 1506 w, 1484 m, 1469 vw, 1460 vw, 1436 w, 1416 w, 1398 w, 1382 w, 1352 s, 1309 vw, 1279 w, 1267 w, 1254 w, 1221 w, 1197 w, 1181 vw, 1113 m, 1093 w, 1059 vs, 1022 m, 1006 s, 964 w, 951 w, 927 m, 847 m, 801 s, 793 s, 784 m, 774 m, 747 w, 734 w, 713 w, 657 s, 647 m, 637 m, 617 w, 510 w, 494 w, 476 vw, 463 w, 434 m cm^−1^. UV-Vis (CHCl_3_): λ_max_ (log ε) = 316 nm (4.10).

Compound **4***:* To a solution of compound **3** (416 mg, 0.924 mmol), Pd(PPh_3_)_4_ (25 mg, 0.022 mmol, 2.3 mol%), and CuI (25 mg, 0.13 mmol, 14 mol%) in degassed ^i^ Pr_2_NH (40 mL) was added ethynyltriisopropylsilane (0.40 mL, 1.8 mmol, 2.0 equiv.) was added under inert atmosphere and the resulting mixture was stirred at rt for 12 h in the dark. Upon the addition of CH_2_Cl_2_ (80 mL), the crude product was washed with saturated aqueous NH_4_Cl (80 mL) and water (3 × 80 mL). The combined aqueous layers were extracted by fresh CH_2_Cl_2_ (20 mL) and the combined organic phases were dried over anhydrous MgSO_4_ and evaporated to dryness in vacuum. Purification by column chromatography on silica gel (hexane-acetone-ether 90:5:5) afforded product **4** (381 mg, 82%) as light brown oil. ^1^H NMR (600 MHz, acetone-*d*_6_): 1.15 (s, 21 H), 4.37 (d, *J* = 1.1, 2 H), 4.57 (s, 2 H), 6.60 (t, *J* = 1.1, 1 H), 6.62 (s, 2 H), 7.37–7.39 (m, 2 H), 7.48–7.50 (m, 2 H). ^13^ C NMR (150 MHz, acetone-*d_6_*): 11.99, 18.98, 67.73, 71.68, 90.60, 108.12, 109.62, 111.41, 118.07, 120.32, 120.41, 123.30, 128.55, 132.67, 135.36, 139.78. HRMS (ESI+): for C_25_H_32_ONaS_4_Si calcd. 527.09975, found 527.09969. MS (EI+): 527 (M^+•^, 19), 505 (100), 450 (5), 403 (10). IR (CHCl_3_): 3079 m, 2959 d, 2944 d, 2866 s, 2156 s, 1608 w, 1592 m, 1507 m, 1463 m, 1411 w, 1387 m, 1355 m, 1257 m, 1177 w, 1116 s, 1072 s, 1019 m, 952 w, 883 s, 832 s, 678 m, 644 d, 621 s, 430 m cm^−1^. UV-Vis (CHCl_3_): λ_max_ (log ε) = 256 (4.68), 268 (4.65), 315 nm (4.23).

Compound **5***:* Compound **4** (90 mg, 0.18 mmol) was stirred at rt with Bu_4_NF (70 mg, 0.27 mmol, 1.5 equiv.) in dry and degassed THF (1 mL) for 20 min. Then, CH_2_Cl_2_ (5 mL) was added and the mixture was filtered through a pad of silica gel on a glass frit, which was washed with more CH_2_Cl_2_ (15 mL). After evaporation of the solvents in vacuum, the crude product (cca 60 mg) was used in the next step without purification. A small analytical sample was purified by column chromatography on silica gel (hexane-acetone-ether 80:10:10) to yield orange-yellow crystals. M.p. 99–100 °C (hexane). ^1^ H NMR (600 MHz, acetone-*d_6_*): 3.65 (s, 1 H), 4.38 (d, *J* = 1.1, 2 H), 4.57 (s, 2 H), 6.61 (t, *J* = 1.1, 1 H), 6.62 (d, *J* = 0.5, 2 H), 7.37–7.40 (m, 2 H), 7.47–7.50 (m, 2 H). ^13^ C NMR (150 MHz, acetone-*d_6_*): 67.81, 71.67, 79.19, 84.04, 109.61, 111.42, 118.08, 120.32, 120.42, 122.36, 128.53, 132.72, 135.35, 139.94. HRMS (EI+): for C_16_H_12_OS_4_ calcd. 347.9771, found 347.9774. MS (EI+): 348 (M^+•^, 100), 232 (12), 218 (28), 217 (27), 173 (5), 159 (5), 148 (5), 146 (35), 129 (5), 115 (28), 102 (14), 88 (5), 76 (5). IR (CHCl_3_): 3307 s, 3113 w, 3079 w, 2109 w, 1611 w, 1591 w, 1521 w, 1509 w, 1412 w, 1355 s, 1175 w, 1116 s, 1072 s, 1020 m, 822 d, 643 s, 621 m, 430 m cm^−1^. UV-Vis (CHCl_3_): λ_max_ (log ε) = 242 (4.43), 253 (4.41), 314 nm (4.09).

Compound **1***:* In a Schlenk flask, compound **5** (63 mg, 0.18 mmol), 4-iodophenyl ethanethioate (110 mg, 0.40 mmol, 2.2 equiv.), Pd(PPh_3_)_4_ (8 mg, 0.007 mmol, 4 mol%), and CuI (8 mg, 0.04 mmol, 20 mol%) were dissolved in a degassed mixture of Et_3_N (4 mL) and THF (4 mL) and the resulting mixture was sonicated in an ultrasonic bath at 35 °C for 1 h. Upon the addition of CH_2_Cl_2_ (10 mL), the crude product was washed with saturated aqueous NH_4_Cl (20 mL) and water (3 × 20 mL). The combined aqueous layers were extracted by fresh CH_2_Cl_2_ (10 mL) and the combined organic phases were dried over anhydrous MgSO_4_ and evaporated to dryness in vacuum. Purification by column chromatography on silica gel (CH_2_Cl_2_-hexane 8:2) afforded yellow crystals of product **1** (73 mg, 81%). M.p. 123–124 °C (CH_2_Cl_2_-hexane). ^1^H NMR (600 MHz, acetone-*d_6_*): 2.44 (s, 3 H), 4.39 (d, *J* = 1.2, 2 H), 4.59 (s, 2 H), 6.62 (t, *J* = 1.1, 1H), 6.62 (d, *J* = 0.6, 2 H), 7.42–7.45 (m, 2 H), 7.46–7.49 (m, 2 H), 7.55–7.58 (m, 2 H), 7.60–7.63 (m, 2 H). ^13^C NMR (150 MHz, acetone-*d_6_*): 30.28, 67.84, 71.73, 89.22, 91.53, 118.11, 120.33, 120.43, 122.78, 124.98, 128.67, 129.68, 132.40, 132.82, 135.34, 135.37, 139.97, 139.04. HRMS (ESI+): for C_24_H_18_O_2_NaS_5_ calcd. 520.98025, found 520.98004. MS (ESI+): 498 (M^+^). IR (CHCl_3_): 3079 w, 2218 w, 1705 s, 1610 w, 1592 m, 1514 m, 1485 m, 1398 m, 1178 w, 1118 s, 1017 m, 831s, 815 w, 702 w cm^−1^. UV-Vis (CHCl_3_): λ_max_ (log ε) = 298 (4.68), 315 nm (4.64).

***1****@AuNPs:* Ligand **1** (10 mg, 0.019 mmol) in THF (5 mL) was treated with methanolic Bu_4_NOH (1M, 30 µL, 1.5 equiv.) and immediately mixed with ethanolic solution of C_12_H_23_S@AuNPs prepared as described in the literature [23] (10 mL). The volume of the mixture was increased to 20 mL by the addition of fresh ethanol and the reaction vessel was wrapped in aluminum foil and left standing at rt for 2 days. Black sediment formed at the bottom of the vial. Yellowish supernatant was removed and the solid product was layered with a 1:1 mixture of ethanol-THF (3 mL) and left to settle again (1–2 h). After removal of the washing solution, the **1**@AuNPs were dispersed in the organic solvent of choice by sonication for 10 min at rt.

## 4. Conclusions

This communication extends our previous reports on the redox chemistry of multiple (two to six) TTF units brought to close proximity by covalent attachment to a molecular backbone [36]. There, we found that only a half of the TTFs became oxidized, thus forming mutual donor–acceptor complexes. Here, we also scrutinized the redox properties of spatially confined TTF units, this time arranged on the surface of 10 nm AuNPs through reversible thiol-gold bonding. In contrast to literature reports on the dip-coating of Pt or Au electrodes for a prolonged amount of time, we used a glassy carbon electrode carefully polished prior to each measurement. The first of the two observed ETs shows a strong electrostatic coupling and adsorptive effects confirming the attachment to AuNPs. The separation of the two redox processes of ligand **1** decreased from 0.478 V (in solution) to 0.304 V (when attached to the AuNPs). Additionally, an extra irreversible oxidation process was observed at ~1.2 V, which is presumably the result of a specific spatial arrangement of the self-assembled ligands, allowing for intermolecular interactions. This communication demonstrates the advantages of the phase-resolved AC voltammetry over traditional electrochemical research only based on the standard voltammetry. Our results confirm that the ligand forming **1**@AuNPs keeps the redox properties of TTF at its terminus. Binding to AuNPs only slightly diminishes the electronic communication between the two redox centers of TTF.

## Data Availability

Not applicable.

## Supplementary Materials

The following supporting information can be downloaded at: https://www.mdpi.com/article/10.3390/molecules27217639/s1, Figure S1: ^1^ H and ^13^ C NMR spectra of compounds 1, 3, 4 and 5; Figure S2: TEM image (magnification 75,000×) with size-distribution histogram (inset) of C_12_H_23_S@AuNPs; Figure S3: The phase-sensitive AC voltammogram of C_12_H_23_S@AuNPs (1.6 Hz, dichloroethane, TBAPF_6_) showing the absence of any faradaic process.

## Sample Availability

Samples are not available from the authors.
