# Supplementary material for: Electrochemistry of Tetrathiafulvalene Ligands Assembled on the Surface of Gold Nanoparticles
Source: Molecules. 2022 Nov 7;27(21):7639. doi: 10.3390/molecules27217639 (PMC9659269; doi:10.3390/molecules27217639)
Supplement: Supplementary file 1 [file molecules-27-07639-s001.zip › molecules-1959713-supplementary.pdf]

Electrochemistry of Tetrathiafulvalene Ligands Assembled onthe Surface of Gold Nanoparticles

[1] Institute of Organic Chemistry and Biochemistry of the Czech Academy of Sciences, Flemingovo nám. 542/2, 160 00 Prague, Czech Republic

[2] J. Heyrovský Institute of Physical Chemistry of the Czech Academy of Sciences, Dolejškova 2155/3, 182 23 Prague, Czech Republic

* Correspondence: lubomir.pospisil@jh-inst.cas.cz (L.P.); ivo.stary@uochb.cas.cz (I.S.)

^{1}H and ^{13}C NMR spectra of compounds **3**, **4**, **5** and **1**

Compound **3**

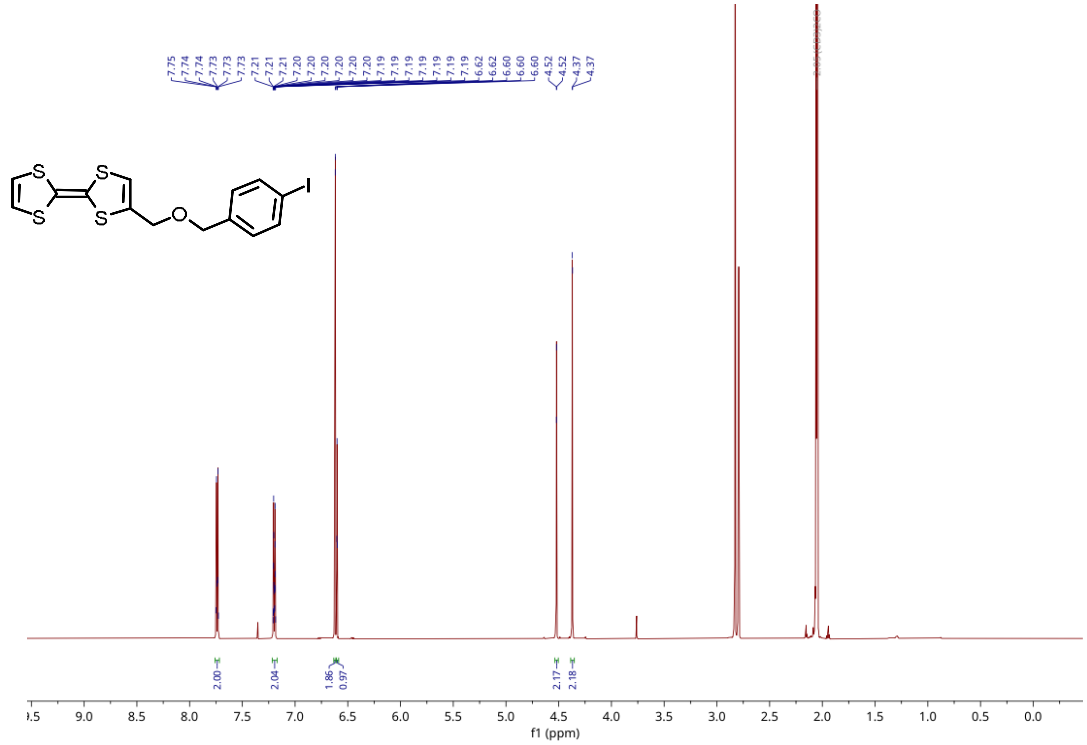

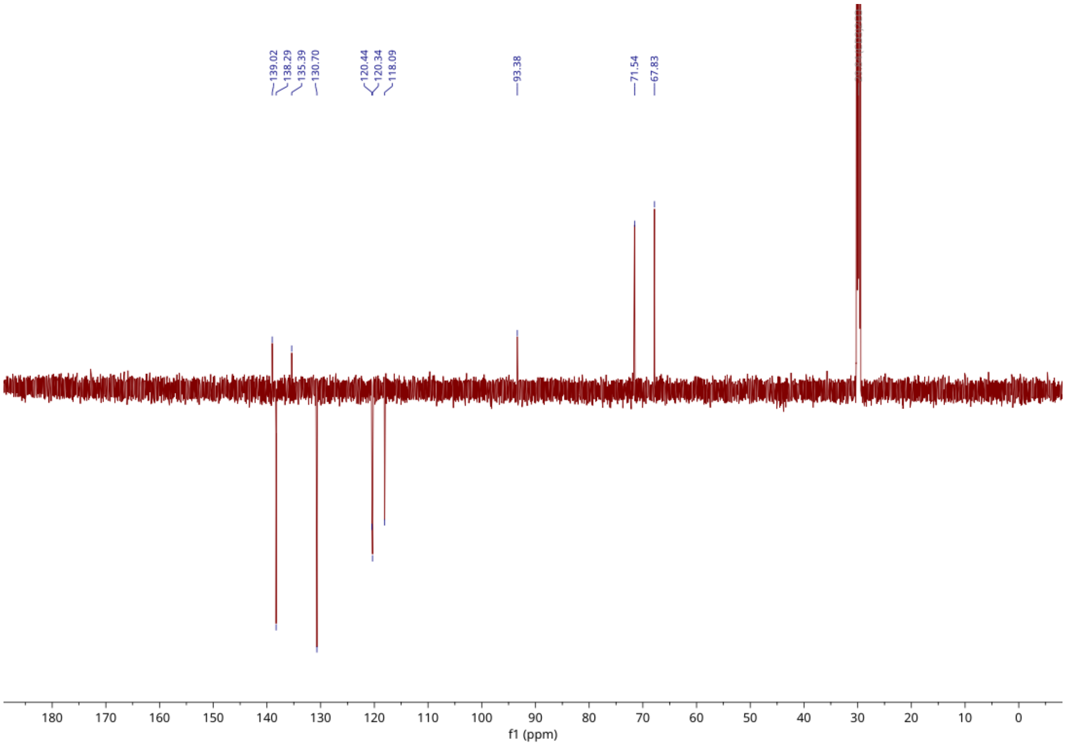

Compound 4

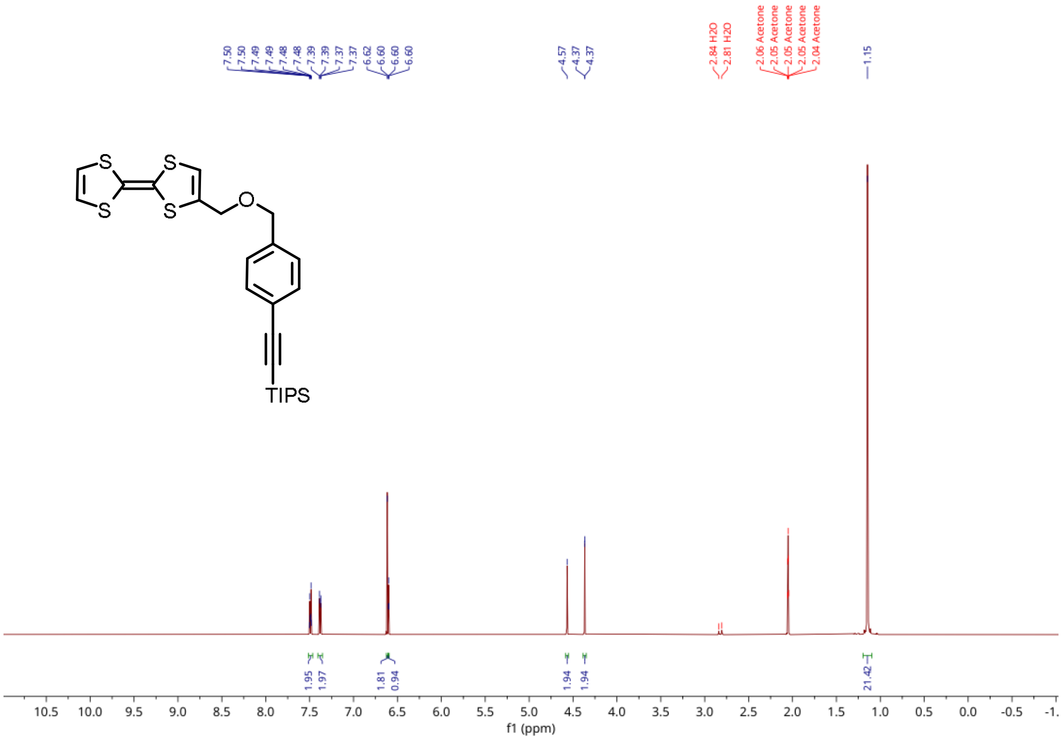
7.50 7.50 7.49 7.49 7.48 7.48 7.39 7.39 7.37 7.37 6.62 6.60 6.60 6.60
4.57 4.37 4.37
2.84 H2O 2.81 H2O
2.06 Acetone 2.05 Acetone 2.05 Acetone 2.05 Acetone 2.04 Acetone
1.15
S
O
TIPS
1.95 1.97 1.81 0.94 1.94 1.94 21.42
10.5 10.0 9.5 9.0 8.5 8.0 7.5 7.0 6.5 6.0 5.5 5.0 4.5 4.0 3.5 3.0 2.5 2.0 1.5 1.0 0.5 0.0 -0.5
f1 (ppm)

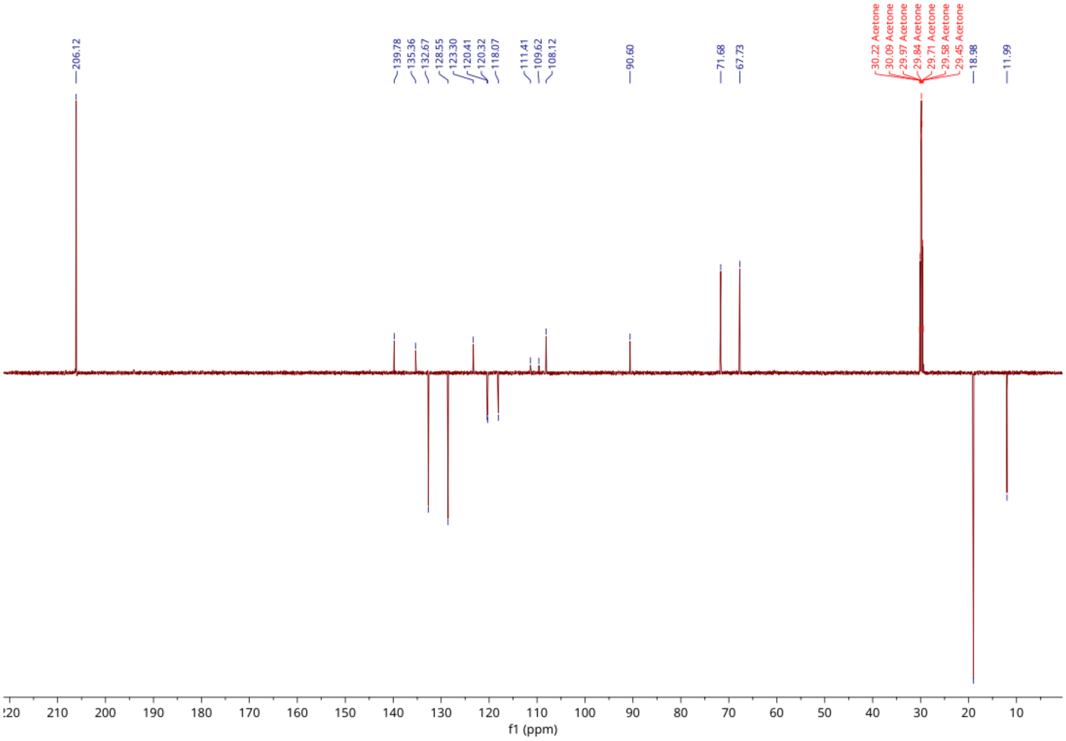
206.12
139.78 135.36 132.67 128.55 123.30 120.41 120.32 118.07 111.41 109.62 108.12
90.60
71.68 67.73
30.22 Acetone 30.09 Acetone 29.97 Acetone 29.84 Acetone 29.71 Acetone 29.58 Acetone 29.45 Acetone
18.98
11.99
210 200 190 180 170 160 150 140 130 120 110 100 90 80 70 60 50 40 30 20 10
f1 (ppm)

Compound 5

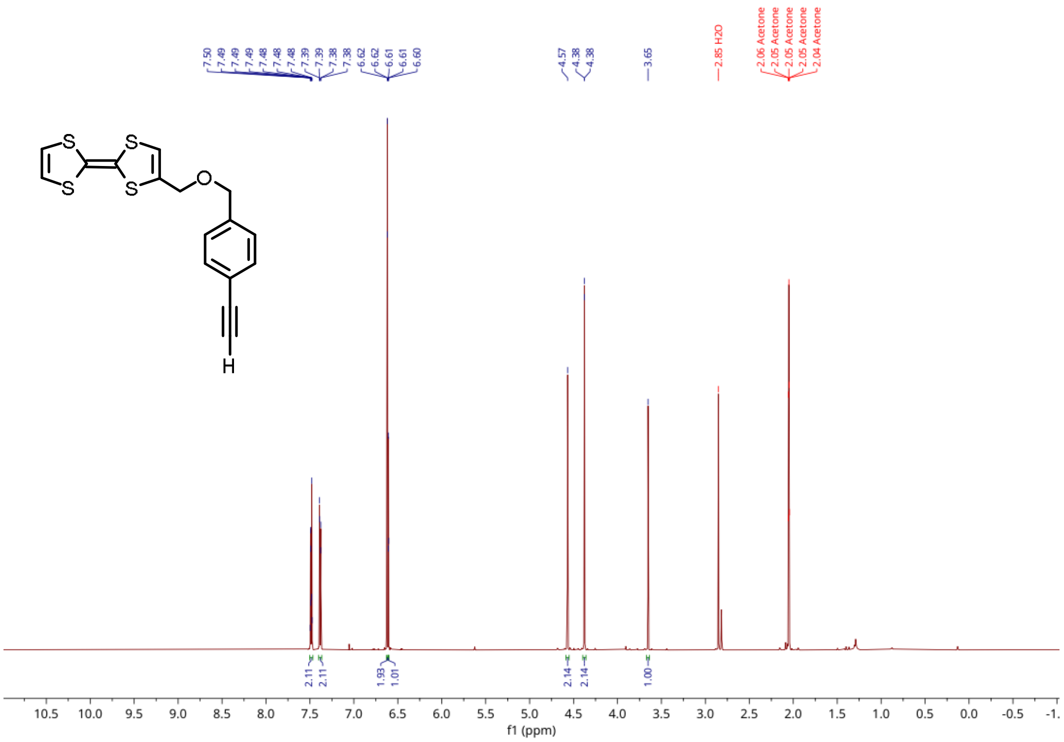
7.50
7.49
7.49
7.49
7.48
7.48
7.48
7.39
7.39
7.38
7.38
6.62
6.62
6.61
6.61
6.60
4.57
4.38
4.38
3.65
2.85 H2O
2.06 Acetone
2.05 Acetone
2.05 Acetone
2.05 Acetone
2.04 Acetone
S
S
S
S
O
H
2.11
2.11
1.93
1.01
2.14
2.14
1.00
10.5
10.0
9.5
9.0
8.5
8.0
7.5
7.0
6.5
6.0
5.5
5.0
4.5
4.0
3.5
3.0
2.5
2.0
1.5
1.0
0.5
0.0
-0.5
f1 (ppm)

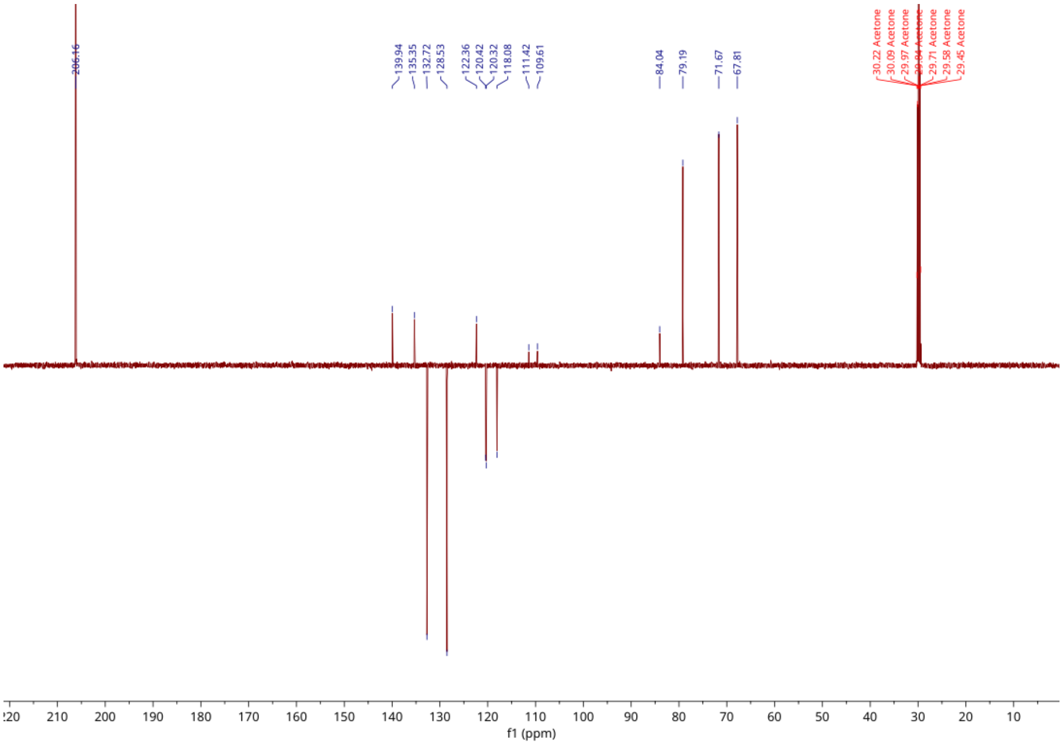
206.16
139.94
135.35
132.72
128.53
122.36
120.42
120.32
118.08
111.42
109.61
84.04
79.19
71.67
67.81
30.22 Acetone
30.09 Acetone
29.97 Acetone
29.71 Acetone
29.58 Acetone
29.45 Acetone
210
200
190
180
170
160
150
140
130
120
110
100
90
80
70
60
50
40
30
20
10
f1 (ppm)

Compound 1

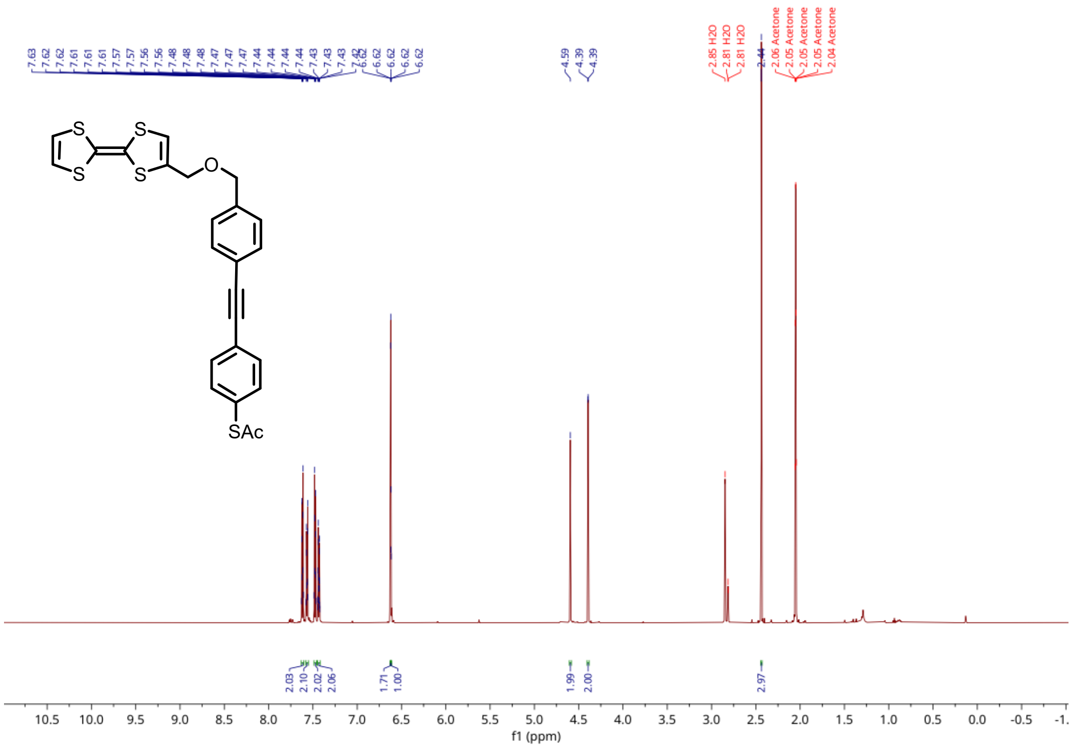

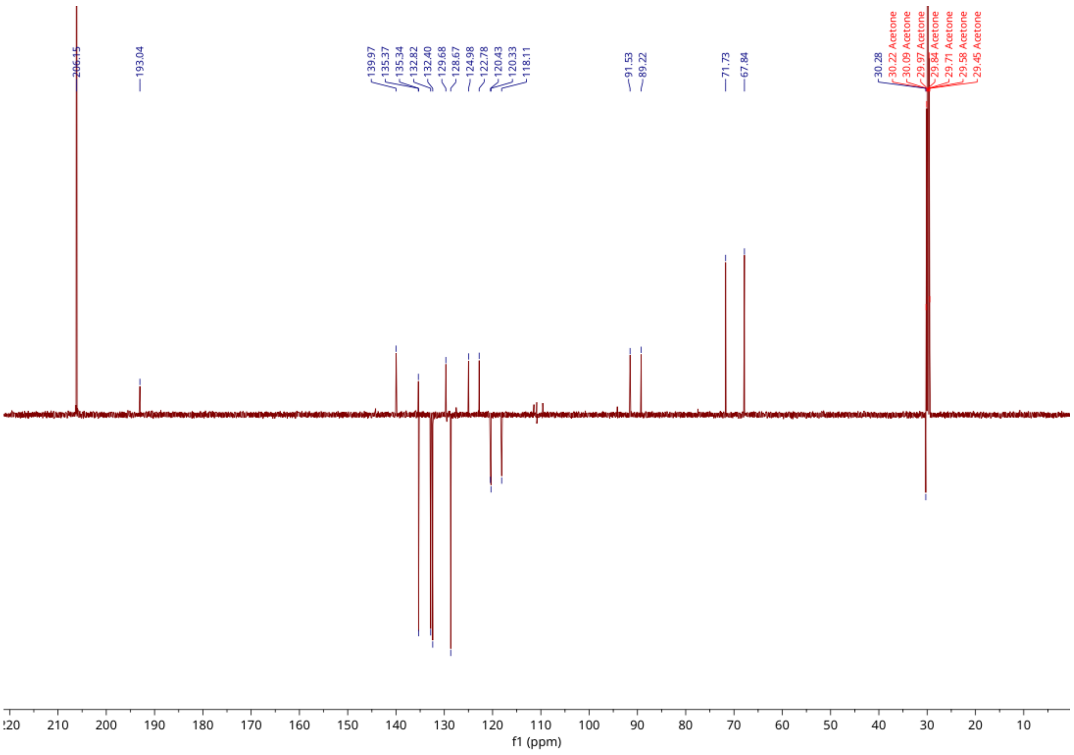

Figure S1 Identification compounds by spectroscopy.

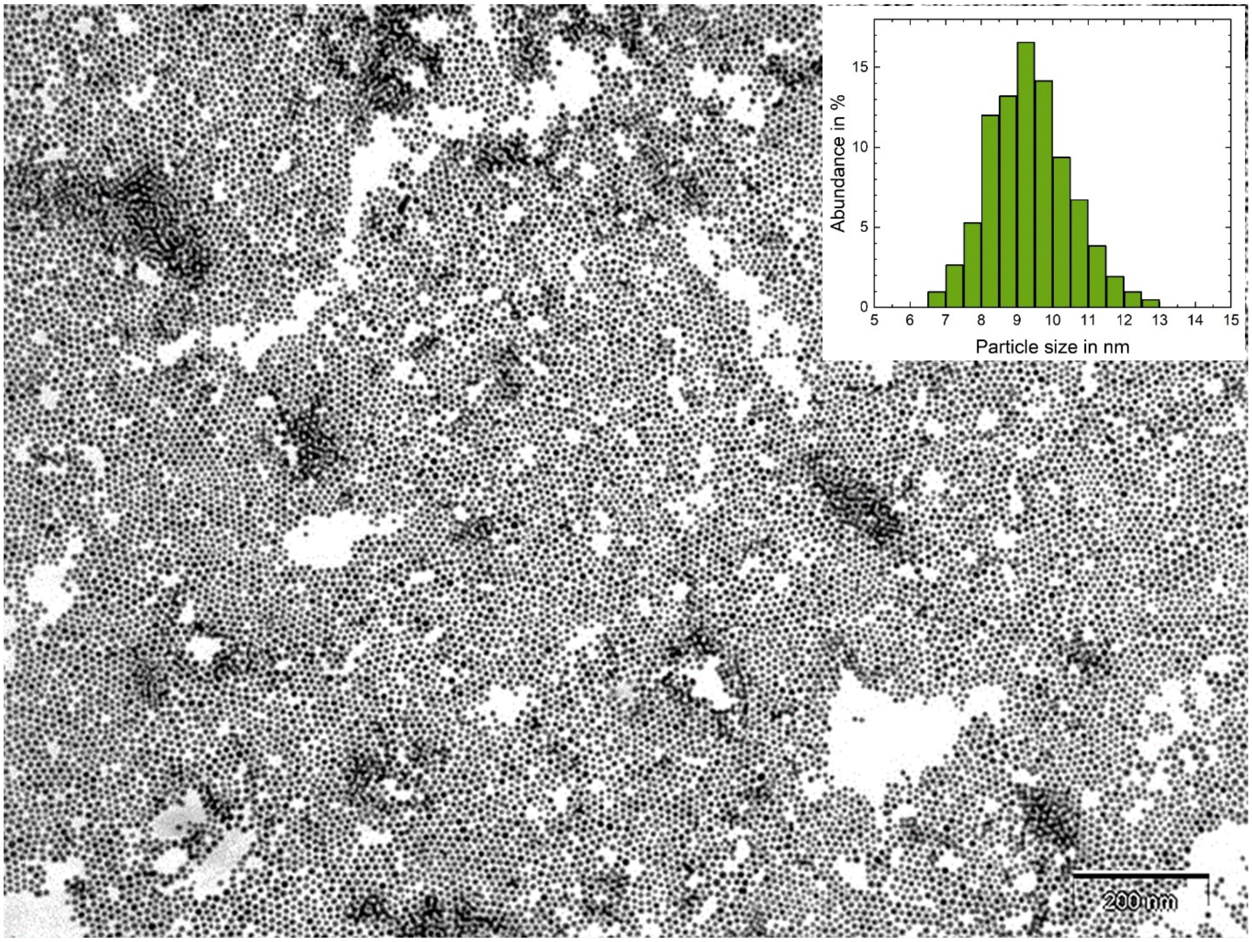

Figure S2: TEM image (magnification 75 000x) with size-distribution histogram (inset) of $C_{12}H_{23}S$@AuNP.

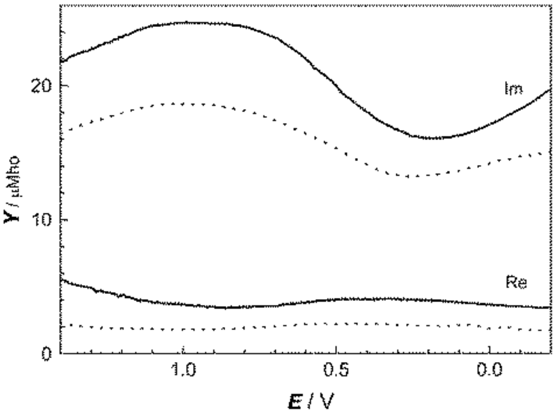

Figure S3: The phase-sensitive AC voltammogram of $C_{12}H_{23}S$@AuNP (1.6 Hz, dichloroethane, $TBAPF_6$) showing the absence of any faradaic process.
